# Cervical cancer screening knowledge and barriers among women in Addis Ababa, Ethiopia

**DOI:** 10.1371/journal.pone.0216522

**Published:** 2019-05-10

**Authors:** Sefonias Getachew, Eyerusalem Getachew, Muluken Gizaw, Wondimu Ayele, Adamu Addissie, Eva J. Kantelhardt

**Affiliations:** 1 Addis Ababa University, College of Health Sciences, School of Public Health, Addis Ababa, Ethiopia; 2 Institute of Epidemiology Biometry and Informatics, Martin Luther University, Halle, Germany; 3 Addis Ababa University, College of Health Sciences, Department of Psychiatry, Addis Ababa, Ethiopia; 4 Department of Gynecology, Martin Luther University, Halle, Germany; Moi University School of Medicine, KENYA

## Abstract

**Introduction:**

Routine cervical screening has been shown to greatly reduce both the number of new cervical cancers diagnosed each year and the number of deaths resulting from the disease. Nevertheless, cervical screening knowledge and screening uptake is very low in developing countries. In Ethiopia, the coverage of cervical cancer screening is only 1%. In this study, we aimed to assess cervical cancer screening knowledge and barriers for screening uptake among women in Addis Ababa Ethiopia.

**Methods:**

A facility-based cross-sectional study was conducted from February to March 2015 in Addis Ababa, Ethiopia. A total of 520 women were selected by a multi-stage sampling procedure. Interview based questioner was used to collect the data. Descriptive statistics was used to describe the socio-demographic and clinical profiles of the women. Multivariate logistic regression using adjusted odds ratio (AOR) and 95% confidence interval (CI) was used to identify independent predictors for cervical screening knowledge. A p-value of <0.05 was set to determine level of statistical significance.

**Results:**

Among all women, 42.7% had heard of cervical cancer screening and 144 (27.7%) women had adequate knowledge of cervical cancer screening. The mean (±SD) age of women was 27.7 (±5.49) years. In total, a quarter (25%) of eligible women had experience of cervical cancer screening. Not being married (adjusted odds ratio (AOR) = 1.8, 1.1–3.3), having an awareness of cervical cancer (AOR = 5.0, 2.7–9.1) and receiving information from health professionals (AOR = 1.9, 1.1–3.2) were the predictors for good cervical cancer screening knowledge. An absence of symptoms (57%), a lack of knowledge about screening (56.3%) and the lack of a screening service in their living area (42.2%) were the perceived barriers for screening uptake.

**Conclusions:**

Cervical screening knowledge was low among women and less than half had heard of screening. Women also had low experience of screening. The lack of a screening service, the absence of symptoms and not knowing about screening were the perceived reasons for the low uptake. Hence, awareness campaigns and education should be undertaken by health professionals. Access and availability of screening service is also essential to improve screening uptake.

## Introduction

Cervical cancer screening enables the detection of abnormal cervical cells, including precancerous cervical lesions, as well as early stage cervical cancers [1]. Routine cervical screening has been shown to reduce both the incidence and mortality of the disease [2]. However, over 80% of invasive cervical cancers worldwide occur in developing countries, largely as a result of the challenges in establishing effective screening programs [3]. The World Health Organisation estimates that only about 5% of women have been screened for cervical cancer in resource-poor countries, compared to 40–50% in the developed world [4]. In Sub Sharan Africa (SSA), there have been efforts to improve awareness and the availability of cervical screening services. However, the coverage still remains low [5] and the incidence and mortality rates associated with the disease are high in this region [6].

Ethiopia is one of the Sub-Saharan countries in which cervical cancer is the second most frequently diagnosed cancer among women, next to breast cancer [7]. The WHO 2012 report showed that the estimated incidence of cervical cancer in Ethiopia was 17.3%, with a mortality of 16.5% [8]. The age-standardised incidence rate of 26.4 per 100, 000 women was estimated in 2012 [7]. Studies have shown that the practice of screening is followed by knowledge of cervical cancer and screening [9]. However, in Ethiopia, the overall coverage of cervical cancer screening was found to be 0.8% according to the ICO Information Centre on HPV and Cancer 2017[10]. A study from the Northeast part of the country showed that only 57.7% of women had ever heard of cervical cancer, and 51.9% had sufficient knowledge of the disease. Nevertheless, 11% had undergone cervical screening at least once in their lifetime [11]. In Mekelle, in the north of the country, among eligible women, 19.8% had been screened for cervical cancer [12], while in the northwest part of Ethiopia, a study showed that only 14.7% of women with knowledge of cervical cancer screening had undergone cervical cancer screening. The study also found that knowledge of the risk factors, symptoms and preventive options regarding cervical cancer were very low [13]; a study in Addis Ababa found that only 6.5% of the respondents had experienced a Pap smear test [9].

It is documented that several factors contribute to inefficient screening for cervical cancer and determine the stage at presentation among patients with cervical cancer in low income countries [14]. The absence of a national screening system and low access to the service have been reported to contribute to inefficient testing and late diagnosis and treatment [15, 16]. One study also found in the country that women are not screened because of absence of gynaecologic symptoms, don’t know where it is done and wait till get older [17]. Ethiopia has currently only has one cervical cancer treatment centre and doing in explanation of the centres. In consideration of the problem and service limitations, there is a commitment to establish other comprehensive cancer centres in the region. Recently, the Ministry of Health has also launched guidelines for cervical cancer prevention, which aim to provide healthcare providers, implementing partners and other stakeholders involved in the prevention and control of cervical cancer in Ethiopia, with standardised cervical cancer prevention and a controlled health service delivery directive [18].

Cervical cancer is a main public health concern among women in the country as there is a scarcity of information on knowledge of screening, practice and barriers related to the uptake of the service in the country, which are needed for effective program implementation. This aims to reduce the incidence and mortality associated with the disease through early diagnosis and treatment as part of targeted interventions. Therefore, the main aim of this study was to determine knowledge about cervical cancer screening and barriers to the uptake of screening services among women visiting reproductive health service clinics at primary care centres in Addis Ababa, Ethiopia.

## Methods

### Study design and period

A facility-based cross-sectional study was conducted from February to March 2015 in Addis Ababa, Ethiopia. Both quantitative and qualitative methods were employed.

### Study area and population

The study population was reproductive age group women attending antenatal follow-up clinics, family planning and postnatal care services at the primary health centres. Addis Ababa is the capital city and a seat of the African union. Currently, it has a total of 86 functional primary health centres in 10 sub cities of Addis Ababa. The geographical health service coverage in Addis Ababa is 100%.

### Sample size and sampling procedure

#### Quantitative

Sample size was calculated using a single proportion formula with knowledge of cervical cancer screening 19% [11], with the assumption of 95% confidence interval and an estimated precision of 0.05. Based on these assumptions, the sample size was calculated to be 236. Considering the 10% none respondent rate and a design effect of two, the final sample size was 520. A multi-stage sampling technique was used to select women visiting a primary health centre. Initially four sub cities from the total sub cities and then 13 primary health centres were randomly selected out of the 37 health centres in four sub cities of Addis Ababa. A proportional allocation of the sample to the respective health centres were done followed by a systematic sampling procedure to include women in the respective health centres. In the systematic sampling we used day interval (Monday, Wednesday and Friday) and all women coming in these days based on eligibility were included until we achieved the sample size. There is no any difference in all days of the week while women are visiting the facility. Women with known mental illness, women who are in labor and within in critical condition during delivery was excluded from the study.

#### Qualitative

Four focus group discussions (FGDs) were conducted on purposively selected women ranging from 8 to 10 from all selected health centres. A total of 37 women were involved out of 45 women invited to attend the FGDs. The number of FGDs was limited to four based on information saturation.

### Data collection procedure

#### Quantitative

Data were collected using a structured interview-based questionnaire and topic guide. The questionnaire was prepared on a review of previous similar studies performed at the country and at alternative locations [9, 19, 20]. The questionnaire was prepared in English and translated to Amharic (S1 File) and then back to English language to maintain the consistency of the information. Data were collected on socio-demographic variables, knowledge of cervical cancer (with regard to risk factors, symptoms, treatment options and prevention and early detection measures), cervical screening knowledge, the utilisation of cervical screening. The questionnaire was pre-tested in 10% of the total sample and necessary amendments were considered.

#### Qualitative

The FGDs were conducted by experts and the information was recorded. The discussions carried out by principal investigator with the assistance of a note taker. A discussion/topic guide (S2 File) was developed by the principal investigator to conduct the focus group discussions. The discussion was tape recorded and transcribed in the same day of the interview. The transcribed data was then translated to English in the next days. Participants were encouraged to speak and express their ideas freely and describe their experience with cases related to the topic. The main areas of discussion was related to knowledge to cervical cancer screening, experience on cervical screening, what are barriers to screening.

### Data Processing and Analysis

#### Quantitative

The data were entered and cleaned using Epi-info version 7.1 and exported to SPSS Version 21 for further analysis. Descriptive statistics like the frequency, proportion, median and interquartile range were used. The median plus interquartile range was used to classify the scores regarding knowledge of cervical cancer screening. Those who scored greater than or equal to the median value of 1 on cervical cancer screening knowledge questions were considered to have adequate knowledge of cervical cancer screening (S1 Table). Cervical cancer knowledge level was determined based on the questions designed to measure the knowledge level and computed using the median value. Binary logistic regression analysis was used to describe the association between cervical screening knowledge and independent variables with the crude odds ratio (COR) and 95% confidence interval. Variables which had a significant association (p value <0.05) with cervical screening knowledge were entered into multivariate analysis to form independent predictors. Multi-variable logistic analysis using an adjusted odds ratio (AOR) was applied to identify the independent predictors for cervical screening knowledge. Level of significance was considered with a p-value less than 0.05.

#### Qualitative

The qualitative analysis was performed using theme analysis predominantly with open code software. The initial and pattern coding was applied to examine and search for similarities and to identify the basis to explain major themes underlying segments of the data. Finally, the findings were described by thematic areas and complimented to the quantitative findings.

### Ethical approval

The research was approved by Addis Ababa university school of public health Research and ethics committee. Following this, the Addis Ababa health office was informed of the study aims and objectives and permission letter was obtained. Then, written consent was secured from the study subjects through informed consent.

## Results

### Socio-demographic characteristics

A total of 520 women participated in the study. Over one third (187; 36%) of the respondents were between 25 and 29 years old. The mean (±SD) age of participants was 27.7 (±5.49) years. Three hundred and nine (59.4%) of the respondents were Orthodox Christian, while 121 (23.3%) were Muslims. Three hundred and sixty nine (71%) respondents were married and 172 (33.1%) had attended primary school. Only one quarter of the respondents (24.6%) attended college level education and above. The majority 205 (39.4%) were housewives, whereas 131 (25.5%) were private employees and 80 (15.4%) were government employees. More than half of the respondents had a monthly household income below 1000 Ethiopian birr (Table 1).

**Table 1:** The socio demographic characteristics of the women’s in Addis Ababa, Ethiopia 2015.

Characteristics	Frequency	Percentage
**n =**	**520**	
Age		
20–24	157	30.2
25–29	189	36.3
30–34	104	20
35–39	53	10.2
40–44	11	2.1
45–49	6	1.2
Marital status		
Married	369	71.0
Single	120	23.1
Divorced	13	2.5
Separated	10	1.9
Widowed	8	1.5
Religion		
Orthodox	309	59.4
Muslim	121	23.3
Protestant	76	14.6
Catholic	14	2.7
Educational status		
No schooling	77	14.8
Primary schooling	172	33.1
Secondary schooling	142	27.3
College/university	128	24.6
Technical vocational	1	0.2
Occupational status		
Housewife	205	39.4
Private employee	131	25.2
Government employee	80	15.4
Daily laborer	29	5.6
Merchant	40	7.7
Student	35	6.7
Monthly income		
<1000	306	58.8
1000–2000	110	21.2
>2000	104	20.0

### Cervical cancer screening knowledge

Table 2 shows knowledge of cervical screening among respondents. Overall, 144 (27.7%) had adequate knowledge of cervical cancer screening. Among the respondents, 222 (42.7%) had heard of cervical cancer screening; of those, 36 (16.2%) knew types of screening methods but only 35 (6.7%) mentioned the pap smear test. Almost half 104 (46.8%) mentioned the frequency of screening as being once per year and 32 (14.4%) stated that this was once every five years. With regard to the age of cervical cancer screening, approximately half (50.9%) indicated that those aged 25 and above should be screened, 49 (22%) said that elderly women should be screened. Among the respondents who had knowledge of cervical cancer screening, one hundred and twenty four (86.1%) had knowledge of cervical cancer, 65 (45.1%) knew someone diagnosed with cervical cancer and 57 (58.8%) mentioned that health professionals were the source of information for knowledge on cervical cancer screening.

**Table 2:** Cervical screening knowledge level among women in Addis Ababa, Ethiopia 2015.

Variables	Frequency	Percentage
Heard of cervical cancer screening		
Yes	222	42.6
No	298	57.3
Know type of cervical cancer screening		
Yes	36	16.2
No	186	83.8
Frequency of cervical screening		
Once every year	104	46.8
Once every three years	17	7.6
Once every five years	32	14.4
Once every six months	21	9.4
Do not know	48	21.6
Age of screening		
Women who starts sexual intercourse	3	1.4
Women age 25 and above	113	51.0
Women age 30 and above	8	3.6
Women age 18 and above	18	8.0
Elderly women	49	22.0
Do not know	31	14.0

Individuals in the FGDs mentioned that they had heard of cervical cancer screening, but some thought that the test was not available in Ethiopia. There were different misconceptions regarding screening, as some participants believed that cervical cancer screening is a method for screening when a woman faces difficulties bearing children and indicated that it is part of the screening procedure during ANC follow-up. Few individuals have a relatively good understanding of screening. They mentioned that it is a screening test performed at a hospital level for women who are sexually active. However, regarding the procedure, most had no awareness. Some stated that it is done by taking fluid from the uterus and knew that there is a special device for the screening, while others said that it is performed using blood tests and examination of vaginal fluid. Almost all of the participants stated that they had only heard the name cervical cancer screening and that they had no detailed knowledge of it. The use of screening is mentioned by all women and they report the benefits in pregnancy and to child health. All participants were willing to undergo screening if the service was available at the nearest location at a reasonable cost. Some of the participants’ views are stated below.

*“Yes*, *there are many types of screening services which might be there but I don't think it exists here in Ethiopia* and …….*It is screening of cervix for cancer if couples get married and find it difficult to bear children*, *and when symptoms appear" (*A 24 year-old postpartum mother, FGD #1).

*"I heard about it but I don't have deep information*. *I think it is a kind of screening during pregnancy*. *I reason it is a procedure done during pregnancy follow-up and I was screened too*.*"* (A 35 year-old postpartum mother FGD #2).

*“It helps if a women plans to have a baby as she should know her health status for the mother and her baby……It is useful to avoid complications during birth*, *to help us know what we should not do"* (A 32 year-old pregnant mother FGD#3).

*"It is useful*, *can prevent late detection*, *and helps to detect the disease early*. *If it is detected early it may be curable*. *If not detected early*, *the germs will spread fast*. *After that*, *the disease cannot be cured"* (A 27 year-old mother FGD#4)

### Factors associated with cervical cancer screening knowledge

Table 3 describes factors associated with cervical screening knowledge with respect to socio-demographic factors, knowledge of cervical cancer itself, sources of information and knowing someone diagnosed with cervical cancer. In bivariate analysis, socio-demographic factors like education, marital status, occupational status and monthly income (self-reported) showed an association with knowledge of cervical cancer screening. However, the age of the women did not show any relationship.

**Table 3:** Factors associated with cervical cancer screening knowledge among women’s in Addis Ababa, Ethiopia 2015.

Variables	Cervical cancer screening knowledge		COR, 95% CI	AOR, 95% CI
	Yes (%)	No (%)		
Educational status				
No schooling	10 (23.8)	32 (76.2)	1	1
Primary schooling	34 (30.1)	79 (69.9)	1.4(0.7–3.1)	0.9 (0.4–2.1)
Secondary schooling	36 (42.4)	49 (57.6)	2.3(1–5.4)	1.8(0.7–4.4)
College/university	64 (59.3)	44 (40.7)	4.6(2.1–10.4)	1.8(0.7–4.6)
Knowledge on cervical cancer				
Yes	124 (54.4)	104 (45.6)	5.9(3.4–10.3)**	5.018(2.7–9.1)**
No	20 (16.7)	100 (83.3)	1	1
Know someone diagnosed with cervical cancer				
Yes	65 (54.2)	55 (45.8)	2.3(1.4–3.5)**	1.2(0.7–2.1)
No	79 (34.6)	149 (65.4)	1	1
Source of information from health professionals				
Yes	57 (58.8)	40 (41.2)	2.7(1.6–4.3)**	1.9(1.1–3.2)*
No	87 (34.7)	164 (65.3)	1	1
Marital status				
Married	91(38.2)	147 (61.8)	1	1
Single	47 (56)	37 (44)	2(1.2–3.4) **	1.8(1.1–3.3)*
Separated	2 (18.2)	9 (81.8)	0.4(0.1–1.7)	0.4(0.1–2.2)
Divorced	2 (22.2)	7 (77.8)	0.5(0.1–2.3)	0.3(0.1–1.9)
Widowed	2 (33.3)	4 (66.7)	0.8(0.1–4.5)	0.9(0.1–6.1)
Monthly income (Birr)				
<1000	63 (33.3)	126 (66.7)	1	1
1000–2000	34 (46.6)	39 (53.4)	1.7(1–3)*	1.4(0.7–2.8)
>2000	47 (54.7)	39 (45.3)	2.4(1.4–4)**	1.4(0.8–2.6)

NB.

* P-value < 0.05

** p-value < 0.001 COR = Crude odds ratio, AOR = Adjusted odds ratio

The provision of information by health professionals, having adequate knowledge of cervical cancer and knowing someone who has been diagnosed with cervical cancer were other factors that are significantly associated with adequate knowledge of cervical cancer screening in the bivariate analysis (Table 3).

In multivariable logistic regression analysis, marital status (being single), having adequate knowledge about the disease (cervical cancer) and women who mentioned health professionals as a source of information were independent predictors for having adequate knowledge on cervical cancer screening. Women who had knowledge of cervical cancer were five times more likely to have knowledge about cervical cancer screening than those who did not (AOR = 5, 95% CI: 2.7–9.0). Women who were single were almost two times more likely to have adequate knowledge of cervical cancer screening than those who were married (AOR = 1.8, 95% CI: 1–3.2), and those with health professionals as a source of information were two times more likely to have knowledge of cervical cancer screening than those who do not mention health professionals as a source of information (AOR = 1.8, 95% CI: 1–3.2) (Table 3).

### Cervical cancer screening practice

Among the eligible women above 35 years old, 25% were screened for cervical cancer in our study. Of these, 66.6% were screened in hospitals and the remaining 33.3% were screened at family guidance association clinics. Almost two thirds (66.7%) of women were screened following initiation by health professionals, while the rest (33.3%) were self-initiated. All of them had only one time exposure for screening.

### Barriers for cervical cancer screening

**Fig 1:**
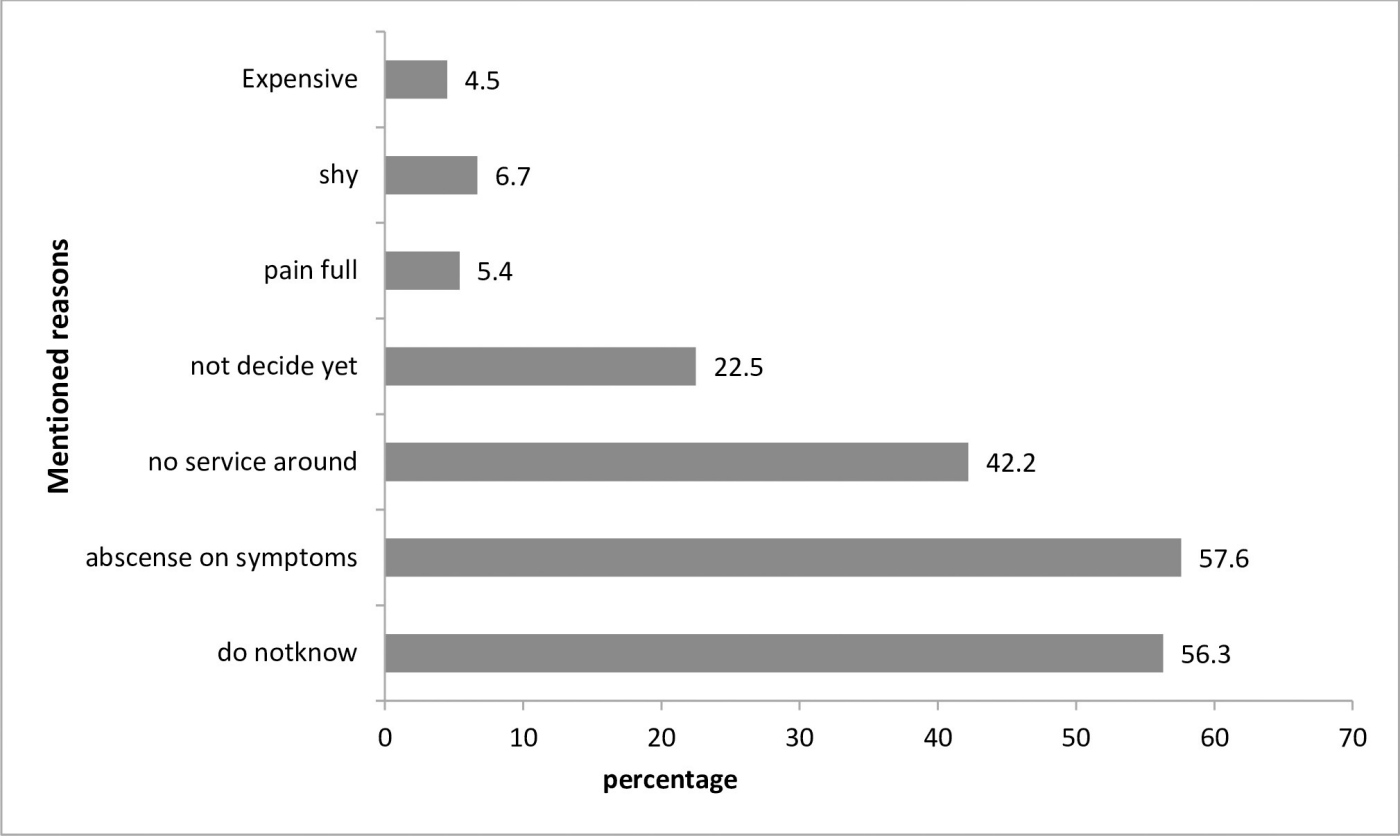
Perceived reasons not to screen among women’s heard about cervical screening, Addis Ababa, Ethiopia 2015.

Women who do not initiate cervical screening give their perceived reasons; in total, 57% mentioned it was due to an absence of signs and symptoms, 56.3% said that they do not know about screening, and 42.2% mentioned that there was no screening service close to them (Fig 1). However, all of the screened women mentioned that cervical cancer can be treated if diagnosed at the earliest stage. The participants also mentioned barriers for undergoing screening practice. In discussions, the main barriers identified were knowledge-related barriers, symptom-related barriers and health system-related barriers.

### Knowledge-related barriers

The participants mentioned that knowledge barriers are the main obstacle. They stated that they lack adequate information regarding cervical cancer itself, about the existence of cervical screening, who is eligible for screening, and where and when they should be screened. They would be happy if they knew detailed information about the issue. Some individuals were even annoyed when the issue was raised. They mentioned that raising the issued by itself was not appropriate since they do not know where the service is available. Also, the fact that the media is not good at informing individuals was highlighted, as women feel that they do not have detailed information. A 26 year-old pregnant mother stated:

*"There is a lack of awareness about the service…. for example, I wanted to check for uterine cancer since I had repeated uterine-related symptoms, but I do not know where to go, who to ask and how much it will cost. I went to the nearby health centre and they gave me some pills, but they did not offer me any tests, so I thought it might not be available in Ethiopia. I still hear about the service in Medias but I do not have any information and I am sure there are many women that have had a similar experience*. *FGD#2"*

### Symptom-related barriers

Another mentioned barrier was a lack of symptoms. The majority of participants mentioned the absence of symptoms as a barrier for screening. They explained that they gave priority to diseases that show symptoms. A 31 year-old postpartum women stated:

*"I live with something that does not have symptoms. For diseases that reveal symptoms, first I try to get better by taking different traditional or modern medicines. If the symptoms persist, that is when I seek the help of health professionals since most symptoms disappear with traditional home treatments*. *FGD#4"*

### Health system-related barriers

Participants also mentioned other barriers related to the health system. The majority of individuals said that the service is not available at their nearest health centre and it is not even available at all hospitals. Only selected hospitals provide the service to clients so the availability issue made it difficult for women to undergo screening. They added that not all professionals offer the service and do not push women to undergo screening. If something is initiated by health professionals, they consider it to be good and are willing to practice what health professionals tell them since they believe that they know better. A 29 year-old women stated that:

*"Most screening services are undertaken following initiation by health professionals, but health professionals do not all initiate cervical cancer screening services*.*FGD#1"*

Availability, accessibility and affordability at reasonable prices were some of the barriers in the health system which are considered to influence the uptake of the screening service.

At the end, individuals said that they need to have detailed information about cervical cancer screening and the disease itself. The service should be available at close locations and they stated the importance of more counselling and discussion with health professionals regarding the issue. In this case, one participant (a 30 year-old pregnant mother) raised the scenario of HIV/AIDS prevention as an example. She stated that:

*"There are different education and awareness creation services for HIV, even in bars/restaurants and other places, but no one has talked about cervical cancer for the last few decades. I think house to house education is necessary for women aged 18 and above. If we look at the scenario of HIV some years back, many people know their status when they seek treatment for other diseases. Currently, due to increased awareness, people are screened voluntarily without the manifestation of any symptoms. Therefore, if awareness creation is undertaken, there will be no chance that people cannot be screened for cervical cancer. It is also important to make the service available and accessible at a reasonable price*. *FGD#3"*

## Discussion

The study identified knowledge of cervical screening and perceived barriers for uptake among women in Addis Ababa primary health care settings. The study found that the level of knowledge about cervical cancer screening was 27%. This was slightly higher than in an Addis Ababa study performed at the hospital level in 2008 which found that the overall level of knowledge was 13.6% [9]. This difference might be explained due to the time and setting difference among the studies and also to slight difference in the definition between studies. However, still our study showed that knowledge to cervical screening among women found to be low for the last nine years. In total, 47.7% of individuals who had heard of cervical cancer screening, which is higher than that found in a study in South Africa in 2010, which showed that 33% of women had heard about cervical cancer screening [21]. This difference might be due to the different study settings. Our investigation was a facility-based study, meaning that participants might have a better health-seeking behaviour and may have had contact with health professionals, exposing them to information about cervical screening.

Regarding factors associated with knowledge of cervical cancer screening, according to our study, women who were single had a greater knowledge of cervical cancer screening than those who were married. This finding was consistent with that of a Malaysian study [22]. The finding can be explained as single women are more likely to be younger and more exposed to sexual education. In our study, women who were knowledgeable about cervical cancer and those women who mentioned health professionals as their source of information were significantly more knowledgeable with regard to cervical cancer screening. This could be explained by the fact that information gained from health professionals could be comprehensive and more detailed than other sources of information.

In our study of eligible women, only 25% had undergone screening. This is a problem in many African countries. In a study performed in Kenya, it was found that only 22% of respondents had been screened [19], while another study in Tanzania showed that only 14% of the respondents had undergone screening [20]. In Addis Ababa, a study revealed that the level of cervical screening was 6.8% [9]. Our study found a relatively higher screening practice, although the majority of women had no experience of screening. Although all screening practices among different countries showed low levels, the findings of our study showed some changes in screening practice.

However, the reasons mentioned in our study for not undergoing screening included an absence of symptoms and a lack of knowledge. A similar study from Addis Ababa reported that reasons not to undergo screening practice included an absence of gynaecological symptoms (41.2%) and a lack of information about places where screening is performed (32.4%) [17]. Another study in Nigeria showed that a fear of the outcome of screening, a lack of information and public awareness, a lack of health worker requests, the high cost of screening and a lack of personnel at the screening centres were the main reasons for a lack of cervical cancer screening [23]. This was also supported by qualitative findings from the focus group discussions, since a number of participants said that most women did not undergo screening because of an absence of symptoms and a lack of information. They stated that they give priority to diseases that show symptoms.

In addition, focus group participants also mentioned other barriers related to health professionals and health facilities. The majority of individuals said that the service was not available at their nearest health centre and that it is not always available at the hospital level; only selected hospitals provide this service. Therefore, the availability issue made it difficult to practice screening. In fact, fewer than 10 out of 86 public health centres in Addis Ababa offer cervical cancer screening services, including a limited number of Hospitals at the public and private level, for the general population of eligible women.

Our study found that women who received information about cervical cancer from health professionals were more likely to undergo screening for cervical cancer than those who did not mention healthcare professionals as a source of information. Also, knowing someone with cervical cancer was associated with good screening practice. This finding was consistent with a study finding in Gondar, where women knowing someone with cervical cancer were more likely to practice cervical cancer screening.

However, the study has some limitations in using the cross-sectional study design which might have a causative effect between determining factors and knowledge about cervical cancer screening.

## Conclusion

We conclude that knowledge of cervical cancer screening and its practices among eligible women was low. Knowledge on cervical cancer and health professionals being a source of information were associated with adequate knowledge of cervical cancer screening. In contrast, the absence of symptoms, not knowing about the service, a lack of screening services in nearby facilities and a lack of decisions to undergo screening were the perceived reasons not to practice screening among eligible women. Hence, health education and awareness creation regarding cervical cancer screening is very essential to women. Special attention should be given to information about eligible age groups, recommended frequencies and screening procedures used. Cervical cancer survivors would be best included in awareness campaigns since knowing a person with cervical cancer was associated with knowledge about the disease and high levels of screening. Health facilities have to improve the access and availability of the service to women.

## Supporting information

S1 File Quantitative tool. (PDF)

S2 File Qualitative topic guide. (PDF)

S1 Table FGD participants file. (PDF)
