# Supplementary material for: Cervical cancer screening knowledge and barriers among women in Addis Ababa, Ethiopia
Source: PLoS One. 2019 May 10;14(5):e0216522. doi: 10.1371/journal.pone.0216522 (PMC6510425; doi:10.1371/journal.pone.0216522)
Supplement: S1 File — (PDF) [file pone.0216522.s001.pdf]

Questionnaire

Part 1 Socio demographic status

No	Questions	Answers	Skip
101	Age in years	________________	
102	Religion	Orthodox............1<br>Muslim................2<br>Protestant............3<br>Catholic.............4<br>Other.................5	
103	Marital status	single... ....1<br>Married................2<br>Divorce................3<br>Widowed............4<br>separated............5	
104	Educational status	illiterate............1<br>primary.............2<br>secondary...........3<br>college................4<br>other..................5	
105	Occupation	House wife..........................1<br>Private employee................2<br>Farmer................................3<br>Government employee....... 4<br>5.Daily laborer....................5<br>6.Merchant......................... 6<br>7.Student............................7<br>8.Others(specify) 8	
106	Monthly Income in Birr		
107	Do you know anyone with cervical cancer	Yes............1<br>No.............2	
Part 2 Risk exposure among the study subjects			
108	How many children do you have	_______In numbers	
109	Age at first sexual intercourse in years	_______ In years	
110	Did you use any contraceptive methods	yes............1<br>No.............2 →	To114
111	If yes what type?	Oral contraceptive pills..........1<br>Injectables..............................2<br>Norplant ....3<br>Barrier methods....................4<br>Other specify 5	If the answer is other than 1 no go to qustion no `114

112	If the response to the above question is 1, for how long did you use oral contraception?	.............Years	
113	Are you currently using oral contraception?	Yes..................1<br>No...................2	
114	Do you smoke?	Yes..................1<br>No...................2	

Part 3 Knowledge about cervical cancer and screening part

No	Questions	Response	Skip
201	Have you ever heard about cancer	Yes............1<br>No.............2 →	End the question here
202	Have you ever heard about cervical cancer	Yes............1<br>No.............2 →	End the question here
203	Where did you first learn about carcinoma of the cervix<br><br>*Multiple answer possible*	News Media............................1<br>Brochures, posters and other printed materials......................2<br>Health workers........................3<br>Family, friends, neighbors and colleagues........,.......................4<br>Religious leaders.....................5<br>Teachers..................................6<br>Other (please explain).............7	
204	What are the symptoms of carcinoma of the cervix?<br><br>*Multiple answer possible*	Vaginal bleeding...................1<br>foul smelling of Vaginal discharges.............................2<br>Do not know..........................3<br>Other.....................................4	
205	What are the risk factors for cancer of the cervix?<br><br>*Multiple answer possible*	Acquiring HPV virus................1<br>Having multiple sexual partners.....................................2<br>Early sexual intercourse............3<br>Avoid having to many children.....................................4<br>Avoid using oral contraceptives for long time.....................................5<br>Cigarette smoking................6<br>Do not know.........................7<br>Other (please explain).........8	
206	How can a person prevent	through vaccination of HPV	

	getting cancer of the cervix?<br><br>*Multiple answer possible*	vaccine..................................1<br>Avoid multiple sexual partners..................................2<br>Avoid early sexual intercourse.............................3<br>Avoid having to many children..4<br>Avoid using oral contraceptives for long time................................5<br>Quit smoking...............................6<br>Screening for cervical cancer......7<br>Other (please explain)..... ....8<br>Do not know...............................9	
207	Can cancer of the cervix be cured in its earliest stages?	Yes.............1<br>No ....2<br>Don't know 3	
208	How can someone with cancer of the cervix be treated?<br><br>*Multiple answer possible*	Herbal remedies...............1<br>surgery.............................2<br>Specific drugs given by hospital............................3<br>radiotherapy....................4<br>Do not know....................5	
209	How expensive do you think cancer of the cervix treatment is in this country?	It is free of charge...................1<br>It is reasonably priced.............2<br>It is somewhat/moderately expensive................................3<br>It is very expensive.................4<br>Don't know...............................5<br>other(specify) 6	
210	Are there screening procedures to detect premalignant cervical lesion?	Yes.............1<br>No...............2	if no 301
211	How frequent is screening for premalignant cervical lesion done?	Once every year....................1<br>Once every three years..........2<br>Once every 5 years................3<br>Any other(mention)..............4<br>Don't know...........................5	
212	Who should be screened?	Women of 25years and above....................................1	

		Prostitutes.............................2<br>Elderly women......................3<br>Other....................................4<br>Don't know............................5	
213	Do you know procedures used in screening for premalignant cervical lesions	Yes...................1<br>No...................2	
214	Can you mention any of the procedures used in screening for premalignant cervical lesions?<br><br>*Multiple answer possible*	VIA...................1<br>VILI..................2<br>Pap Smear........3<br>other…………4<br>don't know........5	

Part 4. Practice Questions

No	Questions	Response	Skip
401	Have you ever heard of screening	yes....................1<br>No....................2	
402	Have you ever screened for any reproductive health screenings like HIV, STIs....	yes....................1<br>No.....................2	If your answer is NO go to question number 407
403	Have you ever screened for cancer of the cervix	Yes...................1<br>No....................2	If your answer is NO go to question number 407
404	Where did you screen	Hospital(Mention)............1<br><br>private(Mention)...............2<br><br>Health centers. (Mention).....3	
405	What was the indication	Self-initiated....................1<br>offered by the health professionals....................2<br>other(specify) 3	
406	If yes how many times in since you become sexually active	Once...................................1<br>More than once..................2	

407	When was the last time you screened	within the past three years<br>.................1<br>More than three years ago.<br>.................2	
408	If no, why?	It may be painful. ....1<br>I feel shy...............................2<br>I am healthy...........................3<br>My husband would not agree......4<br>I am afraid a screening test would reveal cervical cancer...5<br>it is expensive............................6<br>I am not informed/knowledge................7<br>I haven't just decided.............8<br>other.......................................9	
