# Supplementary material for: Cervical cancer screening knowledge and barriers among women in Addis Ababa, Ethiopia
Source: PLoS One. 2019 May 10;14(5):e0216522. doi: 10.1371/journal.pone.0216522 (PMC6510425; doi:10.1371/journal.pone.0216522)
Supplement: S2 File — (PDF) [file pone.0216522.s002.pdf]

Qualitative Topic guide for the Focus group discussion

1. Have you ever heard of cancer of the uterus?
2. Where did you heard?
3. What do you know about the disease? Symptoms treatment, screening, severity...
4. Have you heard of cervical cancer screening?
5. Do you think screening is important, how?
6. Have you ever screened for any reproductive health screenings, HIV, STIs...., if no why
7. Have you ever think to screen
8. What do you think is the reason not to screen for cervical cancer and any other RH screenings in your community?
9. Are you willing to undergo cervical cancer screening, if no why?
10. What do you recommend for the health facilities regarding cervical cancer screening?
