# Supplementary material for: Cervical cancer screening knowledge and barriers among women in Addis Ababa, Ethiopia
Source: PLoS One. 2019 May 10;14(5):e0216522. doi: 10.1371/journal.pone.0216522 (PMC6510425; doi:10.1371/journal.pone.0216522)
Supplement: S1 Table — (PDF) [file pone.0216522.s003.pdf]

Characteristics of women involved in focus group discussion, Addis Ababa, 2015

Characteristics	Frequency	Percentage
**n**	**37**	**100**
**Marital status**		
Married	30	81
Single	7	18.9
**Occupation**		
Housewife	26	70.2
Employee	7	18,9
Students	4	11.8
**Educational status**		
Primary school	20	54
Secondary school	12	32.4
**Above secondary school**	5	13.5
**Reproductive health service seeking**		
ANC	14	37.8
PNC	7	18.9
Family planning	16	43
